# Supplementary material for: The venom gland transcriptome of Tityus paraguayensis reveals a diverse array of bioactive molecules from the Brazilian Cerrado
Source: PLoS One. 2026 Feb 20;21(2):e0343107. doi: 10.1371/journal.pone.0343107 (PMC12923043; doi:10.1371/journal.pone.0343107)
Supplement: S1 Table — (DOCX) [file pone.0343107.s004.docx]

**Supplementary 4**

| ID | Descrição | Classe funcional | Tamanho | TPM |
| --- | --- | --- | --- | --- |
| TRINITY_DN9979_c0_g1_i1 | RecName: Full=Bradykinin-potentiating peptide; Short=BPP; Short=TdBPP; Flags: Precursor | Antihypertensives | 515 | 13,24 |
| TRINITY_DN9979_c1_g1_i1 | RecName: Full=Bradykinin-potentiating peptide; Short=BPP; Short=TdBPP; Flags: Precursor | Antihypertensives | 225 | 0,37 |
| TRINITY_DN4868_c1_g1_i1 | Hypotensin-1; Anti-hypertensive peptide; Hypotensin I; TsHpt-I; Tityustoxin-14 1; | Antihypertensives | 448 | 3561,79 |
| TRINITY_DN4868_c1_g1_i2 | Hypotensin-1; Anti-hypertensive peptide; Hypotensin I; TsHpt-I; Tityustoxin-14 1; | Antihypertensives | 418 | 10358,58 |
| TRINITY_DN4868_c1_g1_i4 | Hypotensin-1; Anti-hypertensive peptide; Hypotensin I; TsHpt-I; Tityustoxin-14 1; | Antihypertensives | 446 | 9,38 |
| TRINITY_DN4868_c1_g1_i5 | Hypotensin-1; Anti-hypertensive peptide; Hypotensin I; TsHpt-I; Tityustoxin-14 1; | Antihypertensives | 439 | 2186,73 |
| TRINITY_DN4868_c2_g1_i1 | Hypotensin-1; Anti-hypertensive peptide; Hypotensin I; TsHpt-I; Tityustoxin-14 1; | Antihypertensives | 205 | 0 |
| TRINITY_DN18338_c0_g1_i1 | Hypotensin-1; Anti-hypertensive peptide; Hypotensin I; TsHpt-I; Tityustoxin-14 1; | Antihypertensives | 321 | 52,07 |
| TRINITY_DN19411_c0_g1_i1 | C9X4J0.1 Bradykinin-potentiating peptide; TdBPP; Flags: Precursor | Antihypertensives | 587 | 6846,98 |
| TRINITY_DN16229_c0_g1_i1 | C9X4J0.1 Bradykinin-potentiating peptide; TdBPP; Flags: Precursor | Antihypertensives | 206 | 4,89 |
| TRINITY_DN2196_c1_g1_i1 | defensin | Antimicrobial | 417 | 20,27 |
| TRINITY_DN582_c0_g1_i1 | defensin precursor | Antimicrobial | 315 | 36,17 |
| TRINITY_DN22929_c0_g1_i1 | defensin-like protein | Antimicrobial | 434 | 266,5 |
| TRINITY_DN12769_c0_g1_i1 | putative antimicrobial peptide precursor | Antimicrobial | 356 | 18904,74 |
| TRINITY_DN12769_c2_g1_i1 | putative antimicrobial peptide precursor | Antimicrobial | 218 | 0,34 |
| TRINITY_DN12769_c3_g1_i1 | putative antimicrobial peptide precursor | Antimicrobial | 289 | 0,93 |
| TRINITY_DN15860_c1_g1_i1 | putative antimicrobial peptide precursor | Antimicrobial | 248 | 0,3 |
| TRINITY_DN15860_c2_g1_i1 | putative antimicrobial peptide precursor | Antimicrobial | 221 | 0 |
| TRINITY_DN15860_c3_g1_i1 | putative antimicrobial peptide precursor | Antimicrobial | 249 | 3056,88 |
| TRINITY_DN15860_c4_g1_i1 | putative antimicrobial peptide precursor | Antimicrobial | 271 | 0,5 |
| TRINITY_DN15860_c5_g1_i1 | putative antimicrobial peptide precursor | Antimicrobial | 206 | 0,45 |
| TRINITY_DN3832_c0_g1_i1 | putative antimicrobial peptide precursor | Antimicrobial | 559 | 1728,06 |
| TRINITY_DN3832_c0_g1_i2 | putative antimicrobial peptide precursor | Antimicrobial | 1194 | 1409,99 |
| TRINITY_DN1068_c0_g2_i1 | putative defensin Tcis59 | Antimicrobial | 392 | 31,81 |
| TRINITY_DN736_c0_g1_i1 | putative defensin Tcis60 | Antimicrobial | 480 | 217,38 |
| TRINITY_DN8522_c0_g3_i1 | RecName: Full=Defensin BmKDfsin6; Flags: Precursor | Antimicrobial | 615 | 9,1 |
| TRINITY_DN8522_c0_g3_i2 | RecName: Full=Defensin BmKDfsin6; Flags: Precursor | Antimicrobial | 474 | 57,84 |
| TRINITY_DN5020_c0_g1_i8 | RecName: Full=Defensin BmKDfsin6; Flags: Precursor | Antimicrobial | 487 | 53,6 |
| TRINITY_DN5020_c0_g1_i9 | RecName: Full=Defensin BmKDfsin6; Flags: Precursor | Antimicrobial | 460 | 18,34 |
| TRINITY_DN13723_c1_g1_i1 | RecName: Full=Anionic peptide clone 9; Flags: Precursor | Antimicrobial | 216 | 0,4 |
| TRINITY_DN13723_c2_g1_i1 | anionic peptide precursor | Antimicrobial | 267 | 0,77 |
| TRINITY_DN2196_c1_g2_i4 | defensin | Antimicrobial | 553 | 218,32 |
| TRINITY_DN7133_c0_g1_i1 | NMR solution structure of Defensin1 from Centruroides limpidus limpidus | Antimicrobial | 998 | 177,79 |
| TRINITY_DN2805_c0_g1_i1 | Non-disulfide-bridged peptide androcin 18-1 | Antimicrobial | 695 | 6577,64 |
| TRINITY_DN12457_c0_g1_i1 | Non-disulfide-bridged peptide androcin 18-1 | Antimicrobial | 323 | 1,86 |
| TRINITY_DN5020_c0_g2_i1 | putative defensin Tcis59 | Antimicrobial | 267 | 14,59 |
| TRINITY_DN5020_c0_g1_i11 | putative defensin Tcis60 | Antimicrobial | 348 | 2,81 |
| TRINITY_DN5020_c0_g1_i4 | putative defensin Tcis60 | Antimicrobial | 434 | 27,93 |
| TRINITY_DN5020_c0_g1_i6 | putative defensin Tcis60 | Antimicrobial | 512 | 2,43 |
| TRINITY_DN5020_c0_g1_i7 | putative defensin Tcis60 | Antimicrobial | 506 | 2,01 |
| TRINITY_DN1068_c0_g1_i1 | putative defensin Tcis60 | Antimicrobial | 358 | 21,45 |
| TRINITY_DN1068_c0_g3_i2 | putative defensin Tcis60 | Antimicrobial | 393 | 66,07 |
| TRINITY_DN4116_c0_g1_i1 | calcium channel toxin-like peptide-1 | Calcium channel modulator | 1093 | 14,42 |
| TRINITY_DN4116_c0_g1_i2 | calcium channel toxin-like peptide-1 | Calcium channel modulator | 551 | 387,61 |
| TRINITY_DN10306_c0_g1_i1 | U8-agatoxin-Ao1a-like | Calcium channel modulator | 389 | 2,02 |
| TRINITY_DN22914_c0_g1_i1 | U8-agatoxin-Ao1a-like isoform X2 | Calcium channel modulator | 547 | 90,72 |
| TRINITY_DN14241_c1_g1_i1 | calcium channel toxin-like peptide-1 | Calcium channel modulator | 613 | 9595,8 |
| TRINITY_DN14241_c1_g1_i2 | calcium channel toxin-like peptide-1 | Calcium channel modulator | 908 | 65,72 |
| TRINITY_DN15053_c1_g1_i1 | calcium channel toxin-like peptide-1 | Calcium channel modulator | 214 | 5,84 |
| TRINITY_DN2068_c0_g1_i8 | antarease-like Zn-metalloprotease | Metalloprotease | 234 | 3357,25 |
| TRINITY_DN10854_c0_g1_i1 | antarease-like Zn-metalloprotease | Metalloprotease | 203 | 9,77 |
| TRINITY_DN45_c0_g1_i41 | venom metalloproteinase antarease-like TtrivMP_A | Metalloprotease | 502 | 44,97 |
| TRINITY_DN342_c0_g2_i4 | venom metalloproteinase antarease-like TtrivMP_A | Metalloprotease | 262 | 9,36 |
| TRINITY_DN1417_c0_g2_i4 | venom metalloproteinase antarease-like TtrivMP_A | Metalloprotease | 314 | 26,23 |
| TRINITY_DN4340_c0_g1_i1 | venom metalloproteinase antarease-like TtrivMP_A | Metalloprotease | 251 | 10,07 |
| TRINITY_DN4340_c0_g1_i2 | venom metalloproteinase antarease-like TtrivMP_A | Metalloprotease | 282 | 3,74 |
| TRINITY_DN1417_c0_g1_i1 | venom metalloproteinase antarease-like TtrivMP_A | Metalloprotease | 340 | 33,91 |
| TRINITY_DN20969_c0_g1_i1 | A disintegrin and metalloproteinase with thrombospondin motifs 6 | Metalloprotease | 204 | 2,3 |
| TRINITY_DN12264_c0_g1_i1 | A disintegrin and metalloproteinase with thrombospondin motifs 6-like | Metalloprotease | 253 | 1,99 |
| TRINITY_DN7034_c0_g1_i1 | A disintegrin and metalloproteinase with thrombospondin motifs 6-like isoform X2 | Metalloprotease | 299 | 2,49 |
| TRINITY_DN7031_c0_g2_i1 | a disintegrin and metalloproteinase with thrombospondin motifs 9 | Metalloprotease | 407 | 2,26 |
| TRINITY_DN11468_c0_g1_i1 | a disintegrin and metalloproteinase with thrombospondin motifs adt-1 | Metalloprotease | 1495 | 6,14 |
| TRINITY_DN11468_c0_g1_i2 | A disintegrin and metalloproteinase with thrombospondin motifs adt-2-like | Metalloprotease | 1116 | 7,86 |
| TRINITY_DN1602_c0_g1_i25 | antarease-like Zn-metalloprotease | Metalloprotease | 1120 | 15,76 |
| TRINITY_DN45_c0_g1_i1 | antarease-like Zn-metalloprotease | Metalloprotease | 782 | 527,28 |
| TRINITY_DN45_c0_g1_i13 | antarease-like Zn-metalloprotease | Metalloprotease | 539 | 17,6 |
| TRINITY_DN45_c0_g1_i15 | antarease-like Zn-metalloprotease | Metalloprotease | 611 | 93,67 |
| TRINITY_DN45_c0_g1_i19 | antarease-like Zn-metalloprotease | Metalloprotease | 399 | 224,51 |
| TRINITY_DN45_c0_g1_i24 | antarease-like Zn-metalloprotease | Metalloprotease | 373 | 236,13 |
| TRINITY_DN45_c0_g1_i30 | antarease-like Zn-metalloprotease | Metalloprotease | 782 | 681,95 |
| TRINITY_DN45_c0_g1_i42 | antarease-like Zn-metalloprotease | Metalloprotease | 854 | 50,88 |
| TRINITY_DN2893_c0_g1_i1 | antarease-like Zn-metalloprotease | Metalloprotease | 690 | 13,34 |
| TRINITY_DN2893_c0_g1_i2 | antarease-like Zn-metalloprotease | Metalloprotease | 690 | 7,53 |
| TRINITY_DN7625_c0_g1_i1 | antarease-like Zn-metalloprotease | Metalloprotease | 545 | 20,21 |
| TRINITY_DN936_c0_g1_i1 | antarease-like Zn-metalloprotease | Metalloprotease | 932 | 62,35 |
| TRINITY_DN936_c0_g1_i9 | antarease-like Zn-metalloprotease | Metalloprotease | 874 | 34,31 |
| TRINITY_DN21893_c0_g1_i1 | astacin-like metalloprotease toxin 1 | Metalloprotease | 1077 | 7,39 |
| TRINITY_DN4294_c0_g1_i1 | astacin-like metalloprotease toxin 1 | Metalloprotease | 856 | 6,71 |
| TRINITY_DN21345_c0_g1_i1 | metalloserrulase 16 | Metalloprotease | 277 | 3,82 |
| TRINITY_DN2002_c0_g2_i1 | metalloserrulase 16 | Metalloprotease | 256 | 815,51 |
| TRINITY_DN1438_c0_g1_i2 | metalloserrulase 16 | Metalloprotease | 347 | 27,93 |
| TRINITY_DN1438_c0_g1_i4 | metalloserrulase 16 | Metalloprotease | 225 | 16,74 |
| TRINITY_DN1438_c0_g1_i8 | metalloserrulase 16 | Metalloprotease | 347 | 81,16 |
| TRINITY_DN936_c0_g2_i1 | metalloserrulase 16 | Metalloprotease | 348 | 83,54 |
| TRINITY_DN936_c0_g2_i2 | metalloserrulase 16 | Metalloprotease | 348 | 67,29 |
| TRINITY_DN2068_c0_g1_i1 | metalloserrulase 6 | Metalloprotease | 313 | 27,9 |
| TRINITY_DN2068_c0_g1_i10 | metalloserrulase 6 | Metalloprotease | 296 | 10,17 |
| TRINITY_DN2068_c0_g1_i2 | metalloserrulase 6 | Metalloprotease | 298 | 5,2 |
| TRINITY_DN1417_c0_g1_i5 | metalloserrulase 6 | Metalloprotease | 229 | 10,21 |
| TRINITY_DN1417_c0_g2_i2 | metalloserrulase 6 | Metalloprotease | 324 | 7,95 |
| TRINITY_DN1602_c0_g2_i1 | putative metalloprotease Tcis_Metallo_8 | Metalloprotease | 277 | 12,32 |
| TRINITY_DN11267_c0_g1_i1 | venom dipeptidyl peptidase 4 | Metalloprotease | 436 | 1,82 |
| TRINITY_DN1438_c0_g1_i1 | venom metalloproteinase antarease-like TpachMP_A | Metalloprotease | 448 | 18,7 |
| TRINITY_DN1438_c0_g1_i6 | venom metalloproteinase antarease-like TpachMP_A | Metalloprotease | 448 | 2,49 |
| TRINITY_DN17532_c0_g1_i1 | venom metalloproteinase antarease-like TpachMP_A | Metalloprotease | 410 | 2,49 |
| TRINITY_DN1828_c0_g1_i1 | venom metalloproteinase antarease-like TtrivMP_A | Metalloprotease | 1447 | 47,68 |
| TRINITY_DN1828_c0_g1_i2 | venom metalloproteinase antarease-like TtrivMP_A | Metalloprotease | 1447 | 5,12 |
| TRINITY_DN1602_c0_g1_i15 | venom metalloproteinase antarease-like TtrivMP_A | Metalloprotease | 1023 | 49,19 |
| TRINITY_DN1602_c0_g1_i20 | venom metalloproteinase antarease-like TtrivMP_A | Metalloprotease | 1077 | 15,21 |
| TRINITY_DN1602_c0_g1_i22 | venom metalloproteinase antarease-like TtrivMP_A | Metalloprotease | 1009 | 17,81 |
| TRINITY_DN1602_c0_g1_i23 | venom metalloproteinase antarease-like TtrivMP_A | Metalloprotease | 1026 | 0 |
| TRINITY_DN1602_c0_g1_i31 | venom metalloproteinase antarease-like TtrivMP_A | Metalloprotease | 983 | 1,91 |
| TRINITY_DN1602_c0_g1_i35 | venom metalloproteinase antarease-like TtrivMP_A | Metalloprotease | 1006 | 74,29 |
| TRINITY_DN1602_c0_g1_i36 | venom metalloproteinase antarease-like TtrivMP_A | Metalloprotease | 535 | 0,37 |
| TRINITY_DN1602_c0_g1_i5 | venom metalloproteinase antarease-like TtrivMP_A | Metalloprotease | 1009 | 55,13 |
| TRINITY_DN13855_c0_g1_i1 | venom metalloproteinase antarease-like TtrivMP_A | Metalloprotease | 803 | 0,99 |
| TRINITY_DN13855_c0_g1_i2 | venom metalloproteinase antarease-like TtrivMP_A | Metalloprotease | 797 | 16,2 |
| TRINITY_DN203_c0_g1_i1 | venom metalloproteinase antarease-like TtrivMP_A | Metalloprotease | 704 | 573,35 |
| TRINITY_DN203_c0_g1_i2 | venom metalloproteinase antarease-like TtrivMP_A | Metalloprotease | 1005 | 11,48 |
| TRINITY_DN203_c0_g1_i3 | venom metalloproteinase antarease-like TtrivMP_A | Metalloprotease | 922 | 7 |
| TRINITY_DN203_c0_g2_i1 | venom metalloproteinase antarease-like TtrivMP_A | Metalloprotease | 1002 | 579,68 |
| TRINITY_DN203_c0_g2_i2 | venom metalloproteinase antarease-like TtrivMP_A | Metalloprotease | 918 | 9,33 |
| TRINITY_DN250_c0_g1_i10 | venom metalloproteinase antarease-like TtrivMP_A | Metalloprotease | 1661 | 1 |
| TRINITY_DN250_c0_g1_i11 | venom metalloproteinase antarease-like TtrivMP_A | Metalloprotease | 1661 | 64,35 |
| TRINITY_DN250_c0_g1_i12 | venom metalloproteinase antarease-like TtrivMP_A | Metalloprotease | 405 | 19,07 |
| TRINITY_DN250_c0_g1_i15 | venom metalloproteinase antarease-like TtrivMP_A | Metalloprotease | 1661 | 272,6 |
| TRINITY_DN250_c0_g1_i16 | venom metalloproteinase antarease-like TtrivMP_A | Metalloprotease | 1661 | 342,42 |
| TRINITY_DN250_c0_g1_i3 | venom metalloproteinase antarease-like TtrivMP_A | Metalloprotease | 1661 | 367,85 |
| TRINITY_DN250_c0_g1_i5 | venom metalloproteinase antarease-like TtrivMP_A | Metalloprotease | 816 | 2173,6 |
| TRINITY_DN250_c0_g1_i7 | venom metalloproteinase antarease-like TtrivMP_A | Metalloprotease | 1661 | 389,75 |
| TRINITY_DN250_c0_g1_i9 | venom metalloproteinase antarease-like TtrivMP_A | Metalloprotease | 291 | 83,48 |
| TRINITY_DN250_c1_g1_i1 | venom metalloproteinase antarease-like TtrivMP_A | Metalloprotease | 316 | 1,19 |
| TRINITY_DN1238_c0_g1_i1 | venom metalloproteinase antarease-like TtrivMP_A | Metalloprotease | 1496 | 7,79 |
| TRINITY_DN1238_c0_g1_i2 | venom metalloproteinase antarease-like TtrivMP_A | Metalloprotease | 1439 | 25,52 |
| TRINITY_DN162_c0_g1_i1 | venom metalloproteinase antarease-like TtrivMP_A | Metalloprotease | 794 | 181,75 |
| TRINITY_DN162_c0_g1_i2 | venom metalloproteinase antarease-like TtrivMP_A | Metalloprotease | 1327 | 50,66 |
| TRINITY_DN9641_c0_g1_i1 | venom metalloproteinase antarease-like TtrivMP_A | Metalloprotease | 1309 | 10,64 |
| TRINITY_DN45_c0_g1_i10 | venom metalloproteinase antarease-like TtrivMP_A | Metalloprotease | 647 | 160,57 |
| TRINITY_DN45_c0_g1_i11 | venom metalloproteinase antarease-like TtrivMP_A | Metalloprotease | 1420 | 21,1 |
| TRINITY_DN45_c0_g1_i18 | venom metalloproteinase antarease-like TtrivMP_A | Metalloprotease | 1567 | 40,24 |
| TRINITY_DN45_c0_g1_i2 | venom metalloproteinase antarease-like TtrivMP_A | Metalloprotease | 773 | 114,86 |
| TRINITY_DN45_c0_g1_i20 | venom metalloproteinase antarease-like TtrivMP_A | Metalloprotease | 1639 | 2,99 |
| TRINITY_DN45_c0_g1_i21 | venom metalloproteinase antarease-like TtrivMP_A | Metalloprotease | 782 | 161,19 |
| TRINITY_DN45_c0_g1_i22 | venom metalloproteinase antarease-like TtrivMP_A | Metalloprotease | 1576 | 81,47 |
| TRINITY_DN45_c0_g1_i27 | venom metalloproteinase antarease-like TtrivMP_A | Metalloprotease | 1071 | 97,87 |
| TRINITY_DN45_c0_g1_i34 | venom metalloproteinase antarease-like TtrivMP_A | Metalloprotease | 1364 | 69,42 |
| TRINITY_DN45_c0_g1_i35 | venom metalloproteinase antarease-like TtrivMP_A | Metalloprotease | 1639 | 35,59 |
| TRINITY_DN45_c0_g1_i39 | venom metalloproteinase antarease-like TtrivMP_A | Metalloprotease | 1364 | 55,75 |
| TRINITY_DN45_c0_g1_i45 | venom metalloproteinase antarease-like TtrivMP_A | Metalloprotease | 1364 | 144,5 |
| TRINITY_DN45_c0_g1_i46 | venom metalloproteinase antarease-like TtrivMP_A | Metalloprotease | 829 | 1,39 |
| TRINITY_DN45_c0_g2_i1 | venom metalloproteinase antarease-like TtrivMP_A | Metalloprotease | 953 | 302,57 |
| TRINITY_DN2068_c0_g1_i11 | venom metalloproteinase antarease-like TtrivMP_A | Metalloprotease | 342 | 27,9 |
| TRINITY_DN2068_c0_g1_i3 | venom metalloproteinase antarease-like TtrivMP_A | Metalloprotease | 216 | 10,12 |
| TRINITY_DN2068_c0_g1_i5 | venom metalloproteinase antarease-like TtrivMP_A | Metalloprotease | 335 | 22,05 |
| TRINITY_DN2068_c0_g1_i6 | venom metalloproteinase antarease-like TtrivMP_A | Metalloprotease | 333 | 25,26 |
| TRINITY_DN2068_c0_g1_i7 | venom metalloproteinase antarease-like TtrivMP_A | Metalloprotease | 216 | 0,82 |
| TRINITY_DN2002_c0_g1_i1 | venom metalloproteinase antarease-like TtrivMP_A | Metalloprotease | 1172 | 14,64 |
| TRINITY_DN2002_c0_g1_i2 | venom metalloproteinase antarease-like TtrivMP_A | Metalloprotease | 632 | 52,93 |
| TRINITY_DN2002_c0_g1_i3 | venom metalloproteinase antarease-like TtrivMP_A | Metalloprotease | 817 | 1,56 |
| TRINITY_DN2002_c0_g1_i4 | venom metalloproteinase antarease-like TtrivMP_A | Metalloprotease | 706 | 27,23 |
| TRINITY_DN2002_c0_g1_i5 | venom metalloproteinase antarease-like TtrivMP_A | Metalloprotease | 1246 | 17,29 |
| TRINITY_DN21644_c0_g1_i1 | venom metalloproteinase antarease-like TtrivMP_A | Metalloprotease | 280 | 0,94 |
| TRINITY_DN2803_c1_g1_i1 | venom metalloproteinase antarease-like TtrivMP_A | Metalloprotease | 377 | 5,37 |
| TRINITY_DN887_c0_g1_i1 | venom metalloproteinase antarease-like TtrivMP_A | Metalloprotease | 1264 | 13,89 |
| TRINITY_DN887_c0_g1_i10 | venom metalloproteinase antarease-like TtrivMP_A | Metalloprotease | 398 | 13,13 |
| TRINITY_DN887_c0_g1_i11 | venom metalloproteinase antarease-like TtrivMP_A | Metalloprotease | 305 | 24,07 |
| TRINITY_DN887_c0_g1_i2 | venom metalloproteinase antarease-like TtrivMP_A | Metalloprotease | 614 | 34,64 |
| TRINITY_DN887_c0_g1_i4 | venom metalloproteinase antarease-like TtrivMP_A | Metalloprotease | 305 | 2,49 |
| TRINITY_DN887_c0_g1_i5 | venom metalloproteinase antarease-like TtrivMP_A | Metalloprotease | 452 | 0,41 |
| TRINITY_DN887_c0_g1_i6 | venom metalloproteinase antarease-like TtrivMP_A | Metalloprotease | 707 | 12,87 |
| TRINITY_DN887_c0_g1_i7 | venom metalloproteinase antarease-like TtrivMP_A | Metalloprotease | 611 | 14,72 |
| TRINITY_DN7625_c0_g1_i2 | venom metalloproteinase antarease-like TtrivMP_A | Metalloprotease | 1294 | 4,97 |
| TRINITY_DN7625_c0_g1_i3 | venom metalloproteinase antarease-like TtrivMP_A | Metalloprotease | 1356 | 0,8 |
| TRINITY_DN7625_c0_g1_i4 | venom metalloproteinase antarease-like TtrivMP_A | Metalloprotease | 1315 | 120,84 |
| TRINITY_DN4002_c0_g1_i1 | venom metalloproteinase antarease-like TtrivMP_A | Metalloprotease | 282 | 60,6 |
| TRINITY_DN4002_c0_g1_i3 | venom metalloproteinase antarease-like TtrivMP_A | Metalloprotease | 392 | 31,28 |
| TRINITY_DN4002_c0_g1_i4 | venom metalloproteinase antarease-like TtrivMP_A | Metalloprotease | 448 | 70,8 |
| TRINITY_DN4002_c0_g1_i6 | venom metalloproteinase antarease-like TtrivMP_A | Metalloprotease | 777 | 5,6 |
| TRINITY_DN4002_c0_g2_i1 | venom metalloproteinase antarease-like TtrivMP_A | Metalloprotease | 425 | 17,49 |
| TRINITY_DN4002_c0_g2_i2 | venom metalloproteinase antarease-like TtrivMP_A | Metalloprotease | 387 | 23,04 |
| TRINITY_DN4002_c0_g3_i1 | venom metalloproteinase antarease-like TtrivMP_A | Metalloprotease | 1354 | 2,44 |
| TRINITY_DN4002_c0_g3_i2 | venom metalloproteinase antarease-like TtrivMP_A | Metalloprotease | 1288 | 55,47 |
| TRINITY_DN7198_c0_g1_i1 | venom metalloproteinase antarease-like TtrivMP_A | Metalloprotease | 681 | 4,53 |
| TRINITY_DN7198_c0_g1_i2 | venom metalloproteinase antarease-like TtrivMP_A | Metalloprotease | 679 | 6,67 |
| TRINITY_DN342_c0_g1_i1 | venom metalloproteinase antarease-like TtrivMP_A | Metalloprotease | 1277 | 31,63 |
| TRINITY_DN342_c0_g1_i3 | venom metalloproteinase antarease-like TtrivMP_A | Metalloprotease | 1307 | 51,68 |
| TRINITY_DN342_c0_g2_i1 | venom metalloproteinase antarease-like TtrivMP_A | Metalloprotease | 1449 | 8,35 |
| TRINITY_DN342_c0_g2_i2 | venom metalloproteinase antarease-like TtrivMP_A | Metalloprotease | 1391 | 47,71 |
| TRINITY_DN342_c0_g2_i3 | venom metalloproteinase antarease-like TtrivMP_A | Metalloprotease | 1561 | 36,18 |
| TRINITY_DN342_c0_g2_i5 | venom metalloproteinase antarease-like TtrivMP_A | Metalloprotease | 1550 | 6,69 |
| TRINITY_DN342_c0_g2_i6 | venom metalloproteinase antarease-like TtrivMP_A | Metalloprotease | 1492 | 33,03 |
| TRINITY_DN1438_c0_g1_i3 | venom metalloproteinase antarease-like TtrivMP_A | Metalloprotease | 229 | 52,48 |
| TRINITY_DN1438_c0_g1_i5 | venom metalloproteinase antarease-like TtrivMP_A | Metalloprotease | 390 | 105,25 |
| TRINITY_DN1438_c0_g1_i7 | venom metalloproteinase antarease-like TtrivMP_A | Metalloprotease | 390 | 19,26 |
| TRINITY_DN1417_c0_g1_i4 | venom metalloproteinase antarease-like TtrivMP_A | Metalloprotease | 262 | 546,49 |
| TRINITY_DN1417_c0_g1_i6 | venom metalloproteinase antarease-like TtrivMP_A | Metalloprotease | 406 | 60,68 |
| TRINITY_DN1417_c0_g1_i7 | venom metalloproteinase antarease-like TtrivMP_A | Metalloprotease | 407 | 14,57 |
| TRINITY_DN1417_c0_g2_i1 | venom metalloproteinase antarease-like TtrivMP_A | Metalloprotease | 575 | 105 |
| TRINITY_DN1417_c0_g2_i3 | venom metalloproteinase antarease-like TtrivMP_A | Metalloprotease | 314 | 8,16 |
| TRINITY_DN1458_c0_g1_i1 | venom metalloproteinase antarease-like TtrivMP_A | Metalloprotease | 441 | 27,85 |
| TRINITY_DN14242_c0_g1_i1 | venom metalloproteinase antarease-like TtrivMP_A | Metalloprotease | 512 | 5,45 |
| TRINITY_DN11423_c0_g1_i1 | venom metalloproteinase antarease-like TtrivMP_A | Metalloprotease | 255 | 2,24 |
| TRINITY_DN936_c0_g1_i10 | venom metalloproteinase antarease-like TtrivMP_A | Metalloprotease | 1015 | 34,65 |
| TRINITY_DN936_c0_g1_i5 | venom metalloproteinase antarease-like TtrivMP_A | Metalloprotease | 505 | 108,49 |
| TRINITY_DN936_c0_g1_i8 | venom metalloproteinase antarease-like TtrivMP_A | Metalloprotease | 1008 | 4,59 |
| TRINITY_DN936_c0_g2_i3 | venom metalloproteinase antarease-like TtrivMP_A | Metalloprotease | 328 | 174,91 |
| TRINITY_DN5845_c0_g1_i1 | venom metalloproteinase antarease-like TtrivMP_A | Metalloprotease | 366 | 21,37 |
| TRINITY_DN5845_c0_g1_i2 | venom metalloproteinase antarease-like TtrivMP_A | Metalloprotease | 366 | 2,73 |
| TRINITY_DN4826_c0_g1_i1 | venom protease-like | Metalloprotease | 1337 | 5,6 |
| TRINITY_DN4826_c0_g1_i4 | venom protease-like | Metalloprotease | 1240 | 15,64 |
| TRINITY_DN11768_c1_g1_i1 | venom protease-like | Metalloprotease | 735 | 3,26 |
| TRINITY_DN3978_c0_g1_i8 | venom protease-like | Metalloprotease | 349 | 2 |
| TRINITY_DN14766_c0_g1_i1 | Xaa-Pro aminopeptidase 1 | Metalloprotease | 2256 | 5,63 |
| TRINITY_DN16455_c0_g1_i1 | neurogenic locus Notch protein | Others | 516 | 3,33 |
| TRINITY_DN3727_c0_g3_i1 | a.superbus venom factor 1 | Others | 1117 | 3,95 |
| TRINITY_DN11016_c0_g1_i1 | a.superbus venom factor 1 | Others | 559 | 4,05 |
| TRINITY_DN3639_c0_g1_i1 | A.superbus venom factor 2-like | Others | 201 | 3,81 |
| TRINITY_DN18911_c0_g1_i1 | Acidic mammalian chitinase | Others | 357 | 1,85 |
| TRINITY_DN3279_c0_g1_i1 | ankyrin repeat domain-containing protein 50-like | Others | 1668 | 6,56 |
| TRINITY_DN856_c0_g1_i1 | ankyrin repeat domain-containing protein 50-like | Others | 284 | 2,96 |
| TRINITY_DN3047_c0_g3_i1 | ankyrin-3-like | Others | 359 | 5,34 |
| TRINITY_DN3047_c0_g4_i1 | ankyrin-3-like | Others | 218 | 9,81 |
| TRINITY_DN5052_c0_g1_i1 | CAP-Gly domain-containing linker protein 3 | Others | 339 | 3,18 |
| TRINITY_DN8212_c0_g1_i1 | CD109 antigen | Others | 214 | 2,46 |
| TRINITY_DN7007_c0_g2_i1 | CD109 antigen | Others | 254 | 2,26 |
| TRINITY_DN979_c0_g1_i1 | CD109 antigen | Others | 267 | 3,84 |
| TRINITY_DN144_c0_g1_i1 | chitin deacetylase 1-like | Others | 2179 | 43,39 |
| TRINITY_DN1584_c0_g1_i1 | CRISP/Allergen/PR-1 | Others | 1840 | 1,79 |
| TRINITY_DN1584_c0_g1_i7 | CRISP/Allergen/PR-1 | Others | 1732 | 0,62 |
| TRINITY_DN6837_c0_g1_i2 | Exploring Cystine Dense Peptide Space to Open a Unique Molecular Toolbox | Others | 543 | 197,77 |
| TRINITY_DN18581_c0_g1_i1 | hemocytin | Others | 224 | 1,85 |
| TRINITY_DN346_c0_g1_i10 | hemocytin-like | Others | 1350 | 85,53 |
| TRINITY_DN346_c0_g1_i11 | hemocytin-like | Others | 1445 | 62,99 |
| TRINITY_DN17070_c0_g1_i1 | Inactive pancreatic lipase-related protein 1 | Others | 232 | 4,78 |
| TRINITY_DN9163_c0_g2_i1 | Neurogenic locus Notch protein | Others | 297 | 2,52 |
| TRINITY_DN9163_c0_g1_i1 | neurogenic locus Notch protein-like | Others | 264 | 1,83 |
| TRINITY_DN17731_c0_g1_i1 | pradykinin-potentiating peptide-like peptide Bpp-1 | Others | 278 | 0,24 |
| TRINITY_DN14330_c1_g1_i1 | pradykinin-potentiating peptide-like peptide Bpp-1 | Others | 221 | 0,71 |
| TRINITY_DN14330_c2_g1_i1 | pradykinin-potentiating peptide-like peptide Bpp-1 | Others | 235 | 0,35 |
| TRINITY_DN1813_c0_g1_i1 | probable chitinase 10 | Others | 413 | 111,89 |
| TRINITY_DN20263_c0_g1_i1 | probable chitinase 10 | Others | 312 | 3,85 |
| TRINITY_DN8253_c0_g1_i1 | probable chitinase 10 | Others | 531 | 6,49 |
| TRINITY_DN23453_c0_g1_i1 | probable chitinase 10 | Others | 259 | 3,53 |
| TRINITY_DN5889_c0_g1_i1 | probable chitinase 10 | Others | 1784 | 29,53 |
| TRINITY_DN8253_c0_g2_i1 | probable chitinase 10 isoform X1 | Others | 255 | 3,08 |
| TRINITY_DN8253_c0_g3_i1 | probable chitinase 10 isoform X2 | Others | 295 | 2,13 |
| TRINITY_DN424_c0_g2_i1 | probable protein S-acyltransferase 23 | Others | 849 | 4,13 |
| TRINITY_DN11679_c0_g1_i1 | protein fem-1 homolog B | Others | 374 | 2,29 |
| TRINITY_DN9760_c0_g1_i1 | protein kinase | Others | 213 | 2,07 |
| TRINITY_DN2540_c0_g1_i1 | Putative venom toxin Ts29; Tityustoxin-29; Flags: Precursor | Others | 956 | 98,37 |
| TRINITY_DN8088_c0_g1_i1 | Venom protein 30.1; Flags: Precursor | Others | 649 | 58,95 |
| TRINITY_DN5302_c0_g1_i2 | toxin CSTX-20 | Others | 389 | 70,47 |
| TRINITY_DN17876_c0_g1_i1 | uncharacterized protein LOC111627489 | Others | 231 | 3,79 |
| TRINITY_DN8597_c0_g2_i1 | uncharacterized protein LOC111635636 | Others | 357 | 2 |
| TRINITY_DN925_c0_g1_i1 | uncharacterized protein LOC111635636 | Others | 1521 | 13 |
| TRINITY_DN7608_c0_g2_i1 | venom allergen 5-like | Others | 441 | 7,05 |
| TRINITY_DN1584_c0_g1_i2 | venom allergen 5-like | Others | 993 | 1,61 |
| TRINITY_DN1584_c0_g1_i4 | venom allergen 5-like | Others | 1020 | 2,55 |
| TRINITY_DN1584_c0_g1_i5 | venom allergen 5-like | Others | 1056 | 0,76 |
| TRINITY_DN1584_c0_g1_i6 | venom allergen 5-like | Others | 1092 | 0,24 |
| TRINITY_DN1778_c0_g3_i1 | venom allergen 5-like | Others | 2606 | 6,62 |
| TRINITY_DN1778_c0_g3_i2 | venom allergen 5-like | Others | 2603 | 11,36 |
| TRINITY_DN18746_c0_g1_i1 | venom peptide BmKAPI-like | Others | 305 | 12,82 |
| TRINITY_DN493_c0_g1_i1 | venom peptide BmKAPI-like | Others | 771 | 457,34 |
| TRINITY_DN5302_c0_g1_i1 | venom protein 164-like | Others | 418 | 2,5 |
| TRINITY_DN21636_c0_g1_i1 | venom protein 164-like | Others | 423 | 5,94 |
| TRINITY_DN21990_c0_g1_i1 | venom protein 164-like | Others | 470 | 6,13 |
| TRINITY_DN18407_c0_g1_i1 | venom protein 29-like | Others | 571 | 124,41 |
| TRINITY_DN18721_c0_g1_i1 | venom protein 29-like | Others | 325 | 4,85 |
| TRINITY_DN9575_c0_g1_i1 | venom protein 29-like | Others | 822 | 45,01 |
| TRINITY_DN19011_c0_g1_i1 | venom protein 29-like | Others | 566 | 12,96 |
| TRINITY_DN22464_c0_g1_i1 | venom protein 302-like | Others | 310 | 8,57 |
| TRINITY_DN2549_c0_g1_i1 | venom protein 302-like | Others | 578 | 117,96 |
| TRINITY_DN2815_c0_g1_i1 | venom protein 302-like | Others | 666 | 1133,24 |
| TRINITY_DN2815_c0_g1_i2 | venom protein 302-like | Others | 744 | 7,22 |
| TRINITY_DN2493_c0_g1_i3 | venom protein 302-like | Others | 503 | 457,84 |
| TRINITY_DN1589_c0_g1_i1 | venom protein 302-like | Others | 661 | 16,16 |
| TRINITY_DN728_c0_g1_i1 | venom protein 302-like | Others | 337 | 63,21 |
| TRINITY_DN728_c0_g2_i1 | venom protein 302-like | Others | 856 | 33,53 |
| TRINITY_DN4179_c0_g1_i1 | venom protein 302-like | Others | 342 | 128,51 |
| TRINITY_DN23446_c0_g1_i1 | venom protein 302-like | Others | 280 | 9,82 |
| TRINITY_DN907_c0_g1_i1 | venom protein 302-like | Others | 518 | 693,85 |
| TRINITY_DN20118_c0_g1_i1 | venom protein 302-like | Others | 346 | 8,9 |
| TRINITY_DN22673_c0_g1_i1 | venom protein 302-like | Others | 246 | 3,32 |
| TRINITY_DN22608_c0_g1_i1 | venom protein 302-like | Others | 709 | 8,24 |
| TRINITY_DN17542_c0_g1_i1 | venom protein 302-like | Others | 564 | 94,8 |
| TRINITY_DN18633_c0_g1_i1 | venom protein 302-like | Others | 665 | 18,95 |
| TRINITY_DN442_c0_g1_i1 | venom protein 302-like | Others | 395 | 0,63 |
| TRINITY_DN442_c0_g1_i2 | venom protein 302-like | Others | 449 | 22,5 |
| TRINITY_DN442_c0_g1_i3 | venom protein 302-like | Others | 404 | 369,69 |
| TRINITY_DN442_c0_g1_i4 | venom protein 302-like | Others | 458 | 19,01 |
| TRINITY_DN442_c0_g1_i6 | venom protein 302-like | Others | 696 | 36,16 |
| TRINITY_DN442_c0_g1_i7 | venom protein 302-like | Others | 642 | 0 |
| TRINITY_DN1015_c0_g1_i1 | venom protein 302-like | Others | 391 | 3,04 |
| TRINITY_DN1015_c0_g1_i2 | venom protein 302-like | Others | 615 | 380,58 |
| TRINITY_DN5076_c0_g1_i1 | venom protein 54.1-like | Others | 414 | 38,14 |
| TRINITY_DN583_c0_g1_i3 | venom toxin | Others | 382 | 4,55 |
| TRINITY_DN715_c0_g1_i1 | venom toxin | Others | 502 | 170,88 |
| TRINITY_DN9767_c0_g2_i1 | venom toxin | Others | 464 | 24,87 |
| TRINITY_DN848_c0_g1_i1 | venom toxin-like peptide | Others | 415 | 58,18 |
| TRINITY_DN848_c0_g1_i2 | venom toxin-like peptide | Others | 415 | 1008,85 |
| TRINITY_DN447_c0_g1_i1 | venom toxin-like peptide | Others | 428 | 29,19 |
| TRINITY_DN447_c0_g1_i3 | venom toxin-like peptide | Others | 399 | 217,72 |
| TRINITY_DN13263_c0_g1_i1 | 85/88 kDa calcium-independent phospholipase A2-like isoform X1 | Others | 268 | 4,32 |
| TRINITY_DN3727_c0_g1_i1 | a.superbus venom factor 1 | Others | 3495 | 28,99 |
| TRINITY_DN3727_c0_g2_i1 | a.superbus venom factor 1 | Others | 665 | 3,75 |
| TRINITY_DN16697_c0_g1_i1 | a.superbus venom factor 1 | Others | 252 | 4,02 |
| TRINITY_DN20637_c0_g1_i1 | allergen Api m 6-like | Others | 527 | 16,43 |
| TRINITY_DN387_c0_g1_i1 | allergen Api m 6-like | Others | 890 | 35,27 |
| TRINITY_DN387_c0_g1_i11 | allergen Api m 6-like | Others | 830 | 1,23 |
| TRINITY_DN387_c0_g1_i12 | allergen Api m 6-like | Others | 886 | 1,36 |
| TRINITY_DN387_c0_g1_i2 | allergen Api m 6-like | Others | 384 | 9,06 |
| TRINITY_DN387_c0_g1_i5 | allergen Api m 6-like | Others | 476 | 11,48 |
| TRINITY_DN387_c0_g1_i6 | allergen Api m 6-like | Others | 890 | 14,96 |
| TRINITY_DN387_c0_g1_i7 | allergen Api m 6-like | Others | 476 | 16,6 |
| TRINITY_DN7372_c0_g2_i1 | allergen Api m 6-like | Others | 687 | 4,87 |
| TRINITY_DN7372_c0_g2_i2 | allergen Api m 6-like | Others | 850 | 159,65 |
| TRINITY_DN4727_c0_g1_i1 | BPTI/Kunitz domain-containing protein | Others | 410 | 4,35 |
| TRINITY_DN226_c0_g1_i1 | Cysteine-rich secretory protein 2 | Others | 1748 | 26,05 |
| TRINITY_DN169_c0_g1_i2 | cysteine-rich secretory protein 2 | Others | 1071 | 55,85 |
| TRINITY_DN11288_c0_g1_i5 | cysteine-rich venom protein LIO1-like | Others | 824 | 15,23 |
| TRINITY_DN11288_c0_g1_i7 | cysteine-rich venom protein LIO1-like | Others | 359 | 8,44 |
| TRINITY_DN20482_c0_g1_i1 | equistatin-like isoform X3 | Others | 466 | 15,41 |
| TRINITY_DN13858_c0_g1_i3 | hypothetical secreted protein | Others | 988 | 36,01 |
| TRINITY_DN1274_c0_g1_i1 | hypothetical secreted protein | Others | 578 | 48,42 |
| TRINITY_DN180_c0_g1_i1 | hypothetical secreted protein | Others | 660 | 111,65 |
| TRINITY_DN84_c0_g1_i11 | hypothetical secreted protein | Others | 1038 | 3,73 |
| TRINITY_DN84_c0_g1_i12 | hypothetical secreted protein | Others | 738 | 161,3 |
| TRINITY_DN84_c0_g1_i2 | hypothetical secreted protein | Others | 863 | 458,96 |
| TRINITY_DN84_c0_g1_i3 | hypothetical secreted protein | Others | 620 | 10,88 |
| TRINITY_DN84_c0_g1_i5 | hypothetical secreted protein | Others | 863 | 4,05 |
| TRINITY_DN84_c0_g1_i8 | hypothetical secreted protein | Others | 515 | 83,41 |
| TRINITY_DN84_c0_g2_i1 | hypothetical secreted protein | Others | 684 | 12,53 |
| TRINITY_DN84_c0_g2_i2 | hypothetical secreted protein | Others | 669 | 96,06 |
| TRINITY_DN20554_c0_g1_i1 | hypothetical secreted protein | Others | 701 | 125,98 |
| TRINITY_DN614_c0_g1_i2 | hypothetical secreted protein | Others | 677 | 638,91 |
| TRINITY_DN614_c0_g1_i3 | hypothetical secreted protein | Others | 980 | 18,99 |
| TRINITY_DN614_c0_g1_i4 | hypothetical secreted protein | Others | 745 | 10,58 |
| TRINITY_DN2286_c0_g1_i2 | hypothetical secreted protein | Others | 513 | 86,45 |
| TRINITY_DN884_c0_g2_i1 | hypothetical secreted protein | Others | 474 | 20 |
| TRINITY_DN884_c0_g3_i1 | hypothetical secreted protein | Others | 630 | 43,21 |
| TRINITY_DN18195_c0_g1_i1 | hypothetical secreted protein | Others | 271 | 1,99 |
| TRINITY_DN3864_c0_g1_i1 | hypothetical secreted protein | Others | 978 | 42,19 |
| TRINITY_DN3810_c0_g1_i1 | hypothetical secreted protein | Others | 589 | 44,76 |
| TRINITY_DN21565_c0_g1_i1 | hypothetical secreted protein | Others | 1277 | 85,02 |
| TRINITY_DN408_c0_g1_i1 | hypothetical secreted protein | Others | 704 | 45,06 |
| TRINITY_DN408_c0_g1_i2 | hypothetical secreted protein | Others | 633 | 49,15 |
| TRINITY_DN2428_c0_g1_i1 | Kunitz-type carboxypeptidase inhibitor Kci-5 | Others | 322 | 10,47 |
| TRINITY_DN14070_c0_g2_i1 | Kunitz-type peptide | Others | 214 | 1,64 |
| TRINITY_DN21875_c0_g1_i1 | kunitz-type protease inhibitor 3 | Others | 209 | 4,33 |
| TRINITY_DN7242_c0_g1_i1 | kunitz-type serine protease inhibitor A | Others | 1101 | 19,73 |
| TRINITY_DN7242_c0_g1_i5 | kunitz-type serine protease inhibitor A | Others | 1088 | 15,72 |
| TRINITY_DN4727_c0_g2_i1 | Kunitz-type serine protease inhibitor bitisilin-3 | Others | 539 | 5 |
| TRINITY_DN2428_c0_g1_i2 | kunitz-type serine protease inhibitor bitisilin-3-like | Others | 322 | 93,31 |
| TRINITY_DN23184_c0_g1_i1 | kunitz-type serine protease inhibitor-like | Others | 322 | 9,3 |
| TRINITY_DN1583_c0_g1_i1 | orphan peptide AbOp-1 | Others | 506 | 1,98 |
| TRINITY_DN1583_c0_g1_i2 | orphan peptide AbOp-1 | Others | 426 | 92,86 |
| TRINITY_DN1130_c0_g1_i1 | orphan peptide AbOp-16 | Others | 639 | 4,24 |
| TRINITY_DN1130_c0_g1_i2 | orphan peptide AbOp-16 | Others | 543 | 29,44 |
| TRINITY_DN884_c0_g4_i1 | orphan peptide AbOp-16 | Others | 618 | 561,27 |
| TRINITY_DN23089_c0_g1_i1 | orphan peptide AbOp-16 | Others | 489 | 16,64 |
| TRINITY_DN2435_c0_g1_i1 | orphan peptide AbOp-24 | Others | 509 | 114,01 |
| TRINITY_DN2435_c0_g1_i2 | orphan peptide AbOp-24 | Others | 546 | 5,26 |
| TRINITY_DN163_c0_g1_i1 | orphan peptide AbOp-9 | Others | 577 | 134,97 |
| TRINITY_DN17407_c0_g1_i1 | orphan peptide AbOp-9 | Others | 589 | 82,27 |
| TRINITY_DN22201_c0_g1_i1 | orphan peptide AbOp-9 | Others | 288 | 6,44 |
| TRINITY_DN19521_c0_g1_i1 | putative neurotoxin LTDF S-18-like protein | Others | 427 | 2,93 |
| TRINITY_DN37_c0_g2_i1 | putative venom toxin Tcis29 | Others | 351 | 94,9 |
| TRINITY_DN7242_c0_g2_i1 | scorpine-like peptide precursor | Others | 762 | 71,43 |
| TRINITY_DN15570_c0_g1_i1 | snake venom 5'-nucleotidase-like | Others | 222 | 1,51 |
| TRINITY_DN4068_c0_g1_i1 | snake venom 5'-nucleotidase-like | Others | 2337 | 18 |
| TRINITY_DN21739_c0_g1_i1 | snake venom 5'-nucleotidase-like isoform X1 | Others | 454 | 3,11 |
| TRINITY_DN467_c0_g1_i1 | snake venom 5'-nucleotidase-like isoform X1 | Others | 2115 | 14,77 |
| TRINITY_DN467_c0_g1_i2 | snake venom 5'-nucleotidase-like isoform X1 | Others | 2784 | 4,85 |
| TRINITY_DN885_c0_g1_i1 | toxin-like protein 14 | Others | 484 | 79,38 |
| TRINITY_DN1532_c0_g1_i1 | toxin-like protein 14 | Others | 684 | 368,22 |
| TRINITY_DN22787_c0_g1_i1 | toxin-like protein 14 | Others | 387 | 4,61 |
| TRINITY_DN19504_c0_g1_i1 | Toxin-like protein 14 like protein | Others | 714 | 499,92 |
| TRINITY_DN22838_c0_g1_i1 | venom allergen 5-like | Others | 1415 | 107,95 |
| TRINITY_DN7608_c0_g1_i1 | venom allergen 5-like | Others | 259 | 4,08 |
| TRINITY_DN3119_c0_g1_i1 | venom allergen 5-like | Others | 1759 | 21,04 |
| TRINITY_DN3119_c0_g1_i2 | venom allergen 5-like | Others | 1455 | 20,85 |
| TRINITY_DN3119_c0_g1_i7 | venom allergen 5-like | Others | 1840 | 6,28 |
| TRINITY_DN3708_c0_g1_i2 | venom carboxylesterase-6 | Others | 1893 | 17,59 |
| TRINITY_DN11016_c0_g1_i2 | venom factor-like isoform X1 | Others | 312 | 1,31 |
| TRINITY_DN417_c0_g2_i1 | venom peptide BmKAPI-like | Others | 332 | 0 |
| TRINITY_DN8226_c0_g1_i1 | venom peptide HtC6Tx3 | Others | 367 | 37,51 |
| TRINITY_DN8226_c0_g1_i2 | venom peptide HtC6Tx3 | Others | 397 | 1,1 |
| TRINITY_DN22702_c0_g1_i1 | venom peptide HtC6Tx3 | Others | 328 | 5,48 |
| TRINITY_DN14921_c1_g1_i1 | venom peptide Htglin gamma | Others | 359 | 7,32 |
| TRINITY_DN844_c0_g1_i1 | venom peptide meuPep26 | Others | 428 | 3294,64 |
| TRINITY_DN13858_c0_g1_i2 | venom peptide meuPep31 | Others | 461 | 5463,89 |
| TRINITY_DN13858_c0_g1_i4 | venom peptide meuPep31 | Others | 917 | 3,2 |
| TRINITY_DN1408_c0_g1_i10 | venom peptide meuPep34 | Others | 588 | 61,01 |
| TRINITY_DN1408_c0_g1_i12 | venom peptide meuPep34 | Others | 443 | 37,52 |
| TRINITY_DN1408_c0_g1_i14 | venom peptide meuPep34 | Others | 529 | 34,29 |
| TRINITY_DN1408_c0_g1_i15 | venom peptide meuPep34 | Others | 423 | 74,38 |
| TRINITY_DN1408_c0_g1_i16 | venom peptide meuPep34 | Others | 423 | 0 |
| TRINITY_DN1408_c0_g1_i2 | venom peptide meuPep34 | Others | 434 | 183,51 |
| TRINITY_DN1408_c0_g1_i3 | venom peptide meuPep34 | Others | 376 | 84,65 |
| TRINITY_DN1408_c0_g1_i4 | venom peptide meuPep34 | Others | 577 | 167,71 |
| TRINITY_DN1408_c0_g1_i5 | venom peptide meuPep34 | Others | 423 | 109,03 |
| TRINITY_DN1408_c0_g1_i6 | venom peptide meuPep34 | Others | 423 | 25,1 |
| TRINITY_DN13568_c0_g1_i1 | venom phosphodiesterase 1 | Others | 1600 | 7,8 |
| TRINITY_DN4286_c0_g1_i1 | venom phosphodiesterase 1 | Others | 1991 | 14,68 |
| TRINITY_DN962_c0_g2_i1 | venom protein | Others | 392 | 4,13 |
| TRINITY_DN1348_c0_g1_i2 | venom protein | Others | 788 | 8,72 |
| TRINITY_DN20786_c0_g1_i1 | venom protein 29-like | Others | 446 | 14,09 |
| TRINITY_DN9575_c0_g1_i2 | venom protein 29-like | Others | 464 | 3,36 |
| TRINITY_DN18070_c0_g1_i1 | venom protein 29-like | Others | 455 | 38,73 |
|  | venom protein 29-like | Others | 566 |  |
| TRINITY_DN914_c0_g1_i4 | venom protein 29-like | Others | 509 | 15,64 |
| TRINITY_DN20071_c0_g1_i1 | venom protein 29-like | Others | 490 | 9,74 |
| TRINITY_DN23439_c0_g1_i1 | venom protein 30.1-like | Others | 227 | 3,94 |
| TRINITY_DN2493_c0_g1_i1 | venom protein 302-like | Others | 690 | 2,35 |
| TRINITY_DN2493_c0_g1_i4 | venom protein 302-like | Others | 688 | 2,46 |
| TRINITY_DN914_c0_g1_i12 | venom protein 7.1-like | Others | 512 | 31,47 |
| TRINITY_DN914_c0_g1_i16 | venom protein 7.1-like | Others | 509 | 8,32 |
| TRINITY_DN914_c0_g1_i6 | venom protein 7.1-like | Others | 509 | 30,71 |
| TRINITY_DN914_c0_g1_i8 | venom protein 7.1-like | Others | 459 | 6,61 |
| TRINITY_DN1247_c0_g1_i3 | venom protein AbVp-1 | Others | 805 | 43,79 |
| TRINITY_DN1152_c0_g1_i1 | venom protein AbVp-14 | Others | 1040 | 752,46 |
| TRINITY_DN15408_c0_g1_i1 | venom protein AbVp-14 | Others | 210 | 2,54 |
| TRINITY_DN1250_c0_g1_i1 | venom protein AbVp-9 | Others | 1252 | 44,73 |
| TRINITY_DN1250_c0_g1_i3 | venom protein AbVp-9 | Others | 1000 | 3726,59 |
| TRINITY_DN15059_c0_g1_i1 | venom protein AbVp-9 | Others | 242 | 2,61 |
| TRINITY_DN66_c0_g1_i1 | venom protein VP3 | Others | 981 | 127,56 |
| TRINITY_DN13601_c1_g1_i1 | venom protein VP3 | Others | 224 | 0 |
| TRINITY_DN15071_c1_g1_i1 | venom protein VP3 | Others | 240 | 0 |
| TRINITY_DN3918_c0_g1_i1 | venom protein VP6 | Others | 977 | 6632,17 |
| TRINITY_DN14921_c0_g1_i1 | venom protein-5 | Others | 615 | 23,99 |
| TRINITY_DN2395_c0_g1_i1 | venom protein-5 | Others | 886 | 169,06 |
| TRINITY_DN23046_c0_g1_i1 | venom protein-5 | Others | 378 | 8,72 |
| TRINITY_DN21898_c0_g1_i1 | venom protein-9 | Others | 270 | 4,26 |
| TRINITY_DN1019_c0_g1_i3 | venom protein-9 | Others | 673 | 2,66 |
| TRINITY_DN583_c0_g1_i1 | venom toxin | Others | 1369 | 605,77 |
| TRINITY_DN583_c0_g1_i2 | venom toxin | Others | 1431 | 20,41 |
| TRINITY_DN18003_c0_g1_i1 | venom toxin-like peptide-6 | Others | 499 | 6,21 |
| TRINITY_DN1229_c0_g1_i1 | pancreatic alpha-amylase-like | Others | 1640 | 35,98 |
| TRINITY_DN6455_c0_g1_i1 | Q5G8B0.1RecName: Full=Anionic peptide clone 9; Flags: Precursor | Others | 390 | 18706,29 |
| TRINITY_DN13723_c0_g1_i1 | Q5G8B0.1RecName: Full=Anionic peptide clone 9; Flags: Precursor | Others | 254 | 2,92 |
| TRINITY_DN1310_c0_g1_i1 | hyaluronidase | Others | 1286 | 45,07 |
| TRINITY_DN8615_c0_g4_i1 | 85/88 kDa calcium-independent phospholipase A2-like isoform X1 | Phospholipase | 574 | 2,95 |
| TRINITY_DN2678_c0_g3_i1 | putative phospholipase B-like 2 | Phospholipase | 1796 | 12,78 |
| TRINITY_DN7151_c0_g1_i1 | group XIIA secretory phospholipase A2 | Phospholipase | 696 | 21,02 |
| TRINITY_DN15851_c0_g1_i1 | scorpine-like peptide precursor | Potassium channel modulator | 237 | 1,38 |
| TRINITY_DN10847_c0_g1_i1 | scorpine-like peptide precursor | Potassium channel modulator | 899 | 4121,18 |
| TRINITY_DN10847_c0_g1_i2 | scorpine-like peptide precursor | Potassium channel modulator | 1347 | 14,18 |
| TRINITY_DN10847_c1_g1_i1 | scorpine-like peptide precursor | Potassium channel modulator | 277 | 0,24 |
| TRINITY_DN16647_c0_g1_i1 | potassium channel blocker AbKTx-5 | Potassium channel modulator | 219 | 0,39 |
| TRINITY_DN1169_c0_g1_i1 | potassium channel blocker AbKTx-5 | Potassium channel modulator | 1094 | 54,85 |
| TRINITY_DN1169_c0_g1_i2 | potassium channel blocker AbKTx-5 | Potassium channel modulator | 1629 | 6,7 |
| TRINITY_DN1169_c0_g1_i3 | potassium channel blocker AbKTx-5 | Potassium channel modulator | 522 | 27,52 |
| TRINITY_DN1169_c0_g1_i5 | potassium channel blocker AbKTx-5 | Potassium channel modulator | 809 | 4802,97 |
| TRINITY_DN1521_c1_g1_i6 | RecName: Full=Peptide TsPep3; AltName: Full=Tityustoxin-13; Short=Ts13; Flags: Precursor | Potassium channel modulator | 309 | 1044,47 |
| TRINITY_DN5168_c0_g1_i1 | Potassium channel toxin alpha-KTx 21.1; Tityustoxin-15; Short=Ts15; Flags: Precursor | Potassium channel modulator | 702 | 472,45 |
| TRINITY_DN315_c0_g1_i1 | Potassium channel toxin alpha-KTx 4.7;T.sigmurus alpha-KTx; Flags: Precursor | Potassium channel modulator | 420 | 409,7 |
| TRINITY_DN315_c0_g2_i1 | Potassium channel toxin alpha-KTx 4.7; T.sigmurus alpha-KTx; Flags: Precursor | Potassium channel modulator | 463 | 359,09 |
| TRINITY_DN1521_c1_g1_i2 | Potassium channel toxin epsilon-KTx 1.1; Tityustoxin-11;Ts11; TsPep1; Flags: Precursor | Potassium channel modulator | 253 | 1718,6 |
| TRINITY_DN1521_c1_g1_i4 | Potassium channel toxin epsilon-KTx 1.1; Tityustoxin-11;Ts11; TsPep1; Flags: Precursor | Potassium channel modulator | 253 | 0 |
| TRINITY_DN1521_c1_g1_i5 | Potassium channel toxin epsilon-KTx 1.1; Tityustoxin-11;Ts11; TsPep1; Flags: Precursor | Potassium channel modulator | 316 | 2562,12 |
| TRINITY_DN1521_c1_g1_i7 | Potassium channel toxin epsilon-KTx 1.1; Tityustoxin-11;Ts11; TsPep1; Flags: Precursor | Potassium channel modulator | 316 | 5823,46 |
| TRINITY_DN1521_c1_g1_i9 | Potassium channel toxin epsilon-KTx 1.1; Tityustoxin-11;Ts11; TsPep1; Flags: Precursor | Potassium channel modulator | 316 | 318,05 |
| TRINITY_DN23277_c0_g1_i1 | RecName: Full=Potassium channel toxin TdiKIK; Short=TdKIK; Flags: Precursor | Potassium channel modulator | 226 | 1,83 |
| TRINITY_DN6631_c0_g1_i1 | RecName: Full=Potassium channel toxin Ts16; AltName: Full=Tityustoxin-16; Flags: Precursor | Potassium channel modulator | 233 | 37,55 |
| TRINITY_DN5116_c0_g1_i12 | Putative potassium channel toxin Ts20; Putative KTx; Tityustoxin-20; Flags: Precursor | Potassium channel modulator | 465 | 11,93 |
| TRINITY_DN5116_c0_g1_i15 | Putative potassium channel toxin Ts20; Putative KTx; Tityustoxin-20; Flags: Precursor | Potassium channel modulator | 512 | 119,18 |
| TRINITY_DN140_c0_g1_i3 | Putative potassium channel toxin Ts20; Putative KTx; Tityustoxin-20; Flags: Precursor | Potassium channel modulator | 383 | 76,77 |
| TRINITY_DN15206_c0_g1_i1 | spondin-1 | Potassium channel modulator | 251 | 2,31 |
| TRINITY_DN23345_c0_g1_i1 | ergtoxin-like protein 1 | Potassium channel modulator | 354 | 62,25 |
| TRINITY_DN6837_c0_g1_i1 | Exploring Cystine Dense Peptide Space to Open a Unique Molecular Toolbox | Potassium channel modulator | 541 | 2,26 |
| TRINITY_DN2304_c1_g1_i1 | K+ toxin-like peptide | Potassium channel modulator | 536 | 1770,74 |
| TRINITY_DN20986_c0_g1_i1 | Chain A, BUTANTOXIN | Potassium channel modulator | 345 | 907,04 |
| TRINITY_DN21238_c0_g1_i1 | kunitz-type U19-barytoxin-Tl1a | Potassium channel modulator | 230 | 5,22 |
| TRINITY_DN22146_c0_g1_i1 | Kunitz-type U19-barytoxin-Tl1a | Potassium channel modulator | 419 | 3,98 |
| TRINITY_DN140_c0_g1_i1 | putative KTx Tcis27 | Potassium channel modulator | 383 | 136,74 |
| TRINITY_DN140_c0_g1_i2 | putative KTx Tcis27 | Potassium channel modulator | 621 | 15,66 |
| TRINITY_DN140_c0_g1_i4 | putative KTx Tcis27 | Potassium channel modulator | 524 | 41,8 |
| TRINITY_DN1521_c1_g1_i1 | Potassium channel toxin epsilon-KTx 1.1; Tityustoxin-11; Ts11; TsPep1; Flags: Precursor | Potassium channel modulator | 316 | 263,87 |
| TRINITY_DN5116_c0_g1_i6 | Putative potassium channel toxin Ts20; Putative KTx; Tityustoxin-20; Flags: Precursor | Potassium channel modulator | 762 | 11,3 |
| TRINITY_DN5116_c0_g1_i8 | Putative potassium channel toxin Ts20; Putative KTx; Tityustoxin-20; Flags: Precursor | Potassium channel modulator | 761 | 51,41 |
| TRINITY_DN18364_c0_g1_i1 | U-scoloptoxin(05)-Sm1a-like isoform X1 | Potassium channel modulator | 766 | 27,33 |
| TRINITY_DN12366_c0_g1_i1 | U-scoloptoxin(11)-Sa2a-like isoform X1 | Potassium channel modulator | 705 | 7,1 |
| TRINITY_DN8539_c0_g1_i1 | U-scoloptoxin(11)-Sm5a-like isoform X1 | Potassium channel modulator | 529 | 3,04 |
| TRINITY_DN18936_c0_g1_i1 | U-scoloptoxin(19)-Tl1a isoform X2 | Potassium channel modulator | 559 | 48,57 |
| TRINITY_DN2726_c0_g1_i1 | venom peptide MmKTx1-like | Potassium channel modulator | 562 | 75,81 |
| TRINITY_DN2726_c0_g1_i2 | venom peptide MmKTx1-like | Potassium channel modulator | 456 | 4,94 |
| TRINITY_DN18682_c0_g1_i1 | venom peptide MmKTx1-like | Potassium channel modulator | 369 | 14,61 |
| TRINITY_DN1062_c0_g1_i1 | venom peptide MmKTx1-like | Potassium channel modulator | 653 | 587,27 |
| TRINITY_DN5116_c0_g1_i17 | A0A218QXE6.1 Putative potassium channel toxin Ts20; Putative KTx; Tityustoxin-20; Flags: Precursor | Potassium channel modulator | 2072 | 1,62 |
| TRINITY_DN1780_c0_g1_i1 | putative NaTx Tcis51 | Sodium channel modulator | 544 | 630,33 |
| TRINITY_DN2518_c1_g1_i1 | Alpha-mammal toxin Ts3; PT-Mice-alpha NaTx3.1; TsIV; Tityustoxin IV-5; Toxin IV-5; TsIV-5; | Sodium channel modulator | 849 | 403,17 |
| TRINITY_DN10353_c0_g1_i2 | RecName: Full=Beta-mammal Tt1g; AltName: Full=T.trivittatus toxin 1 gamma-like; Flags: Precursor | Sodium channel modulator | 378 | 385,99 |
| TRINITY_DN10353_c0_g1_i4 | RecName: Full=Beta-mammal Tt1g; AltName: Full=T.trivittatus toxin 1 gamma-like; Flags: Precursor | Sodium channel modulator | 312 | 3,26 |
| TRINITY_DN10353_c0_g1_i5 | RecName: Full=Beta-mammal Tt1g; AltName: Full=T.trivittatus toxin 1 gamma-like; Flags: Precursor | Sodium channel modulator | 412 | 10863,81 |
| TRINITY_DN2518_c0_g1_i12 | Putative sodium channel toxin Ts17; Putative alpha-NaTx; Tityustoxin-17; Flags: Precursor | Sodium channel modulator | 1677 | 0,38 |
| TRINITY_DN2518_c0_g1_i15 | Putative sodium channel toxin Ts17; Putative alpha-NaTx; Tityustoxin-17; Flags: Precursor | Sodium channel modulator | 1767 | 2,04 |
| TRINITY_DN2518_c0_g1_i2 | Putative sodium channel toxin Ts17; Putative alpha-NaTx; Tityustoxin-17; Flags: Precursor | Sodium channel modulator | 1666 | 2,1 |
| TRINITY_DN2518_c0_g1_i21 | Putative sodium channel toxin Ts17; Putative alpha-NaTx; Tityustoxin-17; Flags: Precursor | Sodium channel modulator | 1756 | 10,8 |
| TRINITY_DN2518_c0_g1_i23 | Putative sodium channel toxin Ts17; Putative alpha-NaTx; Tityustoxin-17; Flags: Precursor | Sodium channel modulator | 1859 | 2,7 |
| TRINITY_DN2518_c0_g1_i28 | Putative sodium channel toxin Ts17; Putative alpha-NaTx; Tityustoxin-17; Flags: Precursor | Sodium channel modulator | 1127 | 876,25 |
| TRINITY_DN2518_c0_g1_i9 | Putative sodium channel toxin Ts17; Putative alpha-NaTx; Tityustoxin-17; Flags: Precursor | Sodium channel modulator | 1138 | 52,1 |
| TRINITY_DN1494_c0_g1_i2 | Putative sodium channel toxin Ts26; Tityustoxin-26; Flags: Precursor | Sodium channel modulator | 450 | 1836,76 |
| TRINITY_DN19692_c0_g1_i1 | Putative sodium channel toxin Ts37; Tityustoxin-37; Flags: Precursor | Sodium channel modulator | 374 | 621,15 |
| TRINITY_DN19008_c0_g1_i1 | RecName: Full=Putative sodium channel toxin Ts38; AltName: Full=Tityustoxin-38; Flags: Precursor | Sodium channel modulator | 455 | 355,63 |
| TRINITY_DN370_c0_g1_i1 | RecName: Full=Putative sodium channel toxin Ts40; AltName: Full=Tityustoxin-40; Flags: Precursor | Sodium channel modulator | 1235 | 127,95 |
| TRINITY_DN370_c0_g1_i3 | RecName: Full=Putative sodium channel toxin Ts40; AltName: Full=Tityustoxin-40; Flags: Precursor | Sodium channel modulator | 1465 | 12,82 |
| TRINITY_DN2489_c0_g1_i1 | RecName: Full=Toxin Tf2; Flags: Precursor | Sodium channel modulator | 711 | 5,12 |
| TRINITY_DN2489_c0_g1_i2 | RecName: Full=Toxin Tf2; Flags: Precursor | Sodium channel modulator | 494 | 4813,61 |
| TRINITY_DN2489_c0_g1_i5 | RecName: Full=Toxin Tf2; Flags: Precursor | Sodium channel modulator | 503 | 1046,75 |
| TRINITY_DN12723_c0_g1_i1 | RecName: Full=Toxin To12; AltName: Full=T-beta* NaTx5.5; Flags: Precursor | Sodium channel modulator | 210 | 722,69 |
| TRINITY_DN2489_c0_g1_i3 | RecName: Full=Toxin To12; AltName: Full=T-beta* NaTx5.5; Flags: Precursor | Sodium channel modulator | 491 | 2826,98 |
| TRINITY_DN2489_c0_g1_i4 | RecName: Full=Toxin To12; AltName: Full=T-beta* NaTx5.5; Flags: Precursor | Sodium channel modulator | 500 | 1350,25 |
| TRINITY_DN8517_c0_g1_i1 | sodium beta toxin | Sodium channel modulator | 730 | 3,78 |
| TRINITY_DN8517_c0_g1_i2 | sodium beta toxin | Sodium channel modulator | 585 | 5750,49 |
| TRINITY_DN951_c0_g1_i1 | sodium channel toxin 18 | Sodium channel modulator | 600 | 560,06 |
| TRINITY_DN951_c0_g1_i2 | sodium channel toxin 18 | Sodium channel modulator | 625 | 10,67 |
| TRINITY_DN23101_c0_g1_i1 | neurotoxin Cex9-like | Sodium channel modulator | 367 | 4,13 |
| TRINITY_DN6917_c0_g1_i4 | neurotoxin Cex9-like | Sodium channel modulator | 600 | 28,9 |
| TRINITY_DN6917_c0_g1_i7 | neurotoxin Cex9-like | Sodium channel modulator | 848 | 148,7 |
| TRINITY_DN6917_c0_g1_i8 | neurotoxin Cex9-like | Sodium channel modulator | 459 | 31,21 |
| TRINITY_DN6917_c0_g1_i9 | neurotoxin Cex9-like | Sodium channel modulator | 1250 | 29,18 |
| TRINITY_DN10353_c0_g1_i7 | neurotoxin Tcl1 precursor | Sodium channel modulator | 308 | 0,09 |
| TRINITY_DN10353_c0_g1_i8 | neurotoxin Tcl1 precursor | Sodium channel modulator | 1219 | 5,37 |
| TRINITY_DN10353_c0_g1_i9 | neurotoxin Tcl1 precursor | Sodium channel modulator | 217 | 8,61 |
| TRINITY_DN10353_c0_g2_i1 | neurotoxin Tcl2 precursor | Sodium channel modulator | 226 | 11,58 |
| TRINITY_DN8304_c0_g1_i1 | putative buthitoxin-like Tcis58 | Sodium channel modulator | 609 | 203,24 |
| TRINITY_DN1494_c0_g1_i1 | Putative sodium channel toxin Ts26; Putative NaTx; ityustoxin-26; Flags: Precursor | Sodium channel modulator | 512 | 15,22 |
| TRINITY_DN3667_c0_g1_i1 | Putative sodium channel toxin Ts39; Tityustoxin-39; Flags: Precursor | Sodium channel modulator | 442 | 5,81 |
| TRINITY_DN3667_c0_g1_i2 | Putative sodium channel toxin Ts39; Tityustoxin-39; Flags: Precursor | Sodium channel modulator | 427 | 196,66 |
| TRINITY_DN21654_c0_g1_i1 | sodium beta toxin | Sodium channel modulator | 202 | 0 |
| TRINITY_DN957_c0_g1_i1 | u24-ctenitoxin-Pn1a | Sodium channel modulator | 686 | 741,73 |
| TRINITY_DN629_c0_g1_i1 | U24-ctenitoxin-Pn1a-like | Sodium channel modulator | 574 | 357,17 |
